# Obesity and weight change during eight years in relation to asthma incidence

**DOI:** 10.1038/s41598-025-20657-8

**Published:** 2025-10-07

**Authors:** Reshed Abohalaka, Selin Ercan, Pinja Ilmarinen, Helena Backman, Linda Ekerljung, Madeleine Rådinger, Bright I. Nwaru, Hannu Kankaanranta

**Affiliations:** 1 Krefting Research Centre, Department of Internal Medicine and Clinical Nutrition, Sahlgrenska Academy, Institute of Medicine, University of Gothenburg, Medicinaregatan 1F, Box 424, 405 30 Gothenburg, Sweden; 2 Department of Respiratory Medicine, Seinäjoki Central Hospital, Seinäjoki, Finland; 3 Faculty of Medicine and Health Technology, Tampere University, Tampere, Finland; 4 Department of Public Health and Clinical Medicine, Umeå University, Umeå, Sweden; 5 Department of Internal Medicine/Respiratory Medicine and Allergology, The Sahlgrenska University Hospital, Gothenburg, Sweden; 6 Wallenberg Centre for Molecular and Translational Medicine, University of Gothenburg, Gothenburg, Sweden

**Keywords:** Asthma, Obesity, Weight change, Risk, BMI, WSAS, Respiratory tract diseases, Risk factors, Epidemiology

## Abstract

Obesity has been associated with increased prevalence of asthma in cross-sectional studies. We aim to examine the relation of obesity and weight change to 8-year asthma incidence in a randomly selected population-based adult cohort. West Sweden asthma study survey was performed in 2008 (18,087 participated) and follow-up in 2016 (12,449 participated). Participants responded to a questionnaire on these two occasions. Obesity was defined as body mass index (BMI) ≥ 30 (kg/m^2^), and overweight as 25 ≤ BMI < 30 (kg/m^2^). Asthma was defined with affirmative answer to the question “Have you been diagnosed with asthma by a physician?“. Asthma incidence was reported per 1000 person-year over the 8-year follow-up period. Among participants without asthma (*N* = 10,769) in 2008, asthma incidence was 1.8 per 1000 person-year. Asthma incidence was higher in 40–60 years old, normal, or overweight participants who gained ≥ 4 BMI (kg/m^2^), or in those who were obese with a stable BMI. Asthma incidence sharply increased in those who gained ≥ 4 BMI (kg/m^2^), especially in women. Additionally, the risk of asthma was significantly higher in a dose-dependent manner among those who gained ≥ 0.5 BMI (kg/m^2^) compared to those with stable, normal weight. The risk of asthma was higher in those who were obese at baseline regardless of their weight change than individuals with stable, normal weight. Our findings highlight the necessity for health care to integrate weight management strategies as a key component of asthma prevention and care, particularly for women in middle age who are overweight or obese.

**Supplementary Information:**

The online version contains supplementary material available at 10.1038/s41598-025-20657-8.

## Introduction

Asthma and obesity are common disorders that pose a significant public health burden^1^. Obesity increases the risk of various comorbidities, including cardiovascular diseases, type 2 diabetes, and respiratory conditions like sleep apnea and asthma^2,3^. In recent years, obesity is documented as a risk factor for asthma, increasing its occurrence and worsening outcomes^4,5^. Obese individuals with asthma often exhibit a distinct phenotype characterized by more severe symptoms, frequent exacerbations, poorer disease control, and the need for high-dose inhaled corticosteroids. This pattern is observed in both children and adults, as shown in cross-sectional and longitudinal studies^5–9^.

Apart from clinical outcomes, many cross-sectional studies have shown an association between obesity and prevalent asthma^10^. Recent cohort studies also revealed that obesity increased the risk of asthma^11–19^, especially in women^13–16,19^. Although there is a connection between obesity and increased incidence of asthma, the association with overweight is less clear. Some studies reported an increased risk^12–15,19^, while others found no connection^11,20^. In addition, many studies^11–13,15–19^ and a systemic review and meta-analysis^21^ suggest a dose-dependent relationship, where the risk of asthma increases with every 5-unit rise in categorical body mass index (BMI). However, findings vary and lack the knowledge on the specific BMI level at which the risk starts to rise.

It is important to note that these studies primarily used single or categorical measurement of BMI, overlooking the continuous nature of weight changes over time. A recent systemic review and meta-analysis report^22^ showed that only four studies examined the association between weight change and the risk of developing asthma^14,19,23,24^. Of these, two focused exclusively on women^14,19^ while three had a restricted age range for participants^14,23,24^, making them less representative of the general population. In addition, no studies analyzed asthma risk across different age groups and weight categories in relation to weight change, potentially masking risks among those who maintained, gained, or lost weight, regardless of their initial age or BMI. Understanding this is crucial, as asthma mechanisms may depend on both age and baseline BMI and weight gain, rather than considering each factor separately^25,26^. Hence, the current study aimed to determine the relation of obesity and weight change to new-onset asthma in an 8-year follow-up of a population-representative adult cohort, considering factors like age, sex, and baseline BMI.

## Methods

Complete details concerning methods appear in an online data supplement.

### Study area and population

**Fig. 1:**
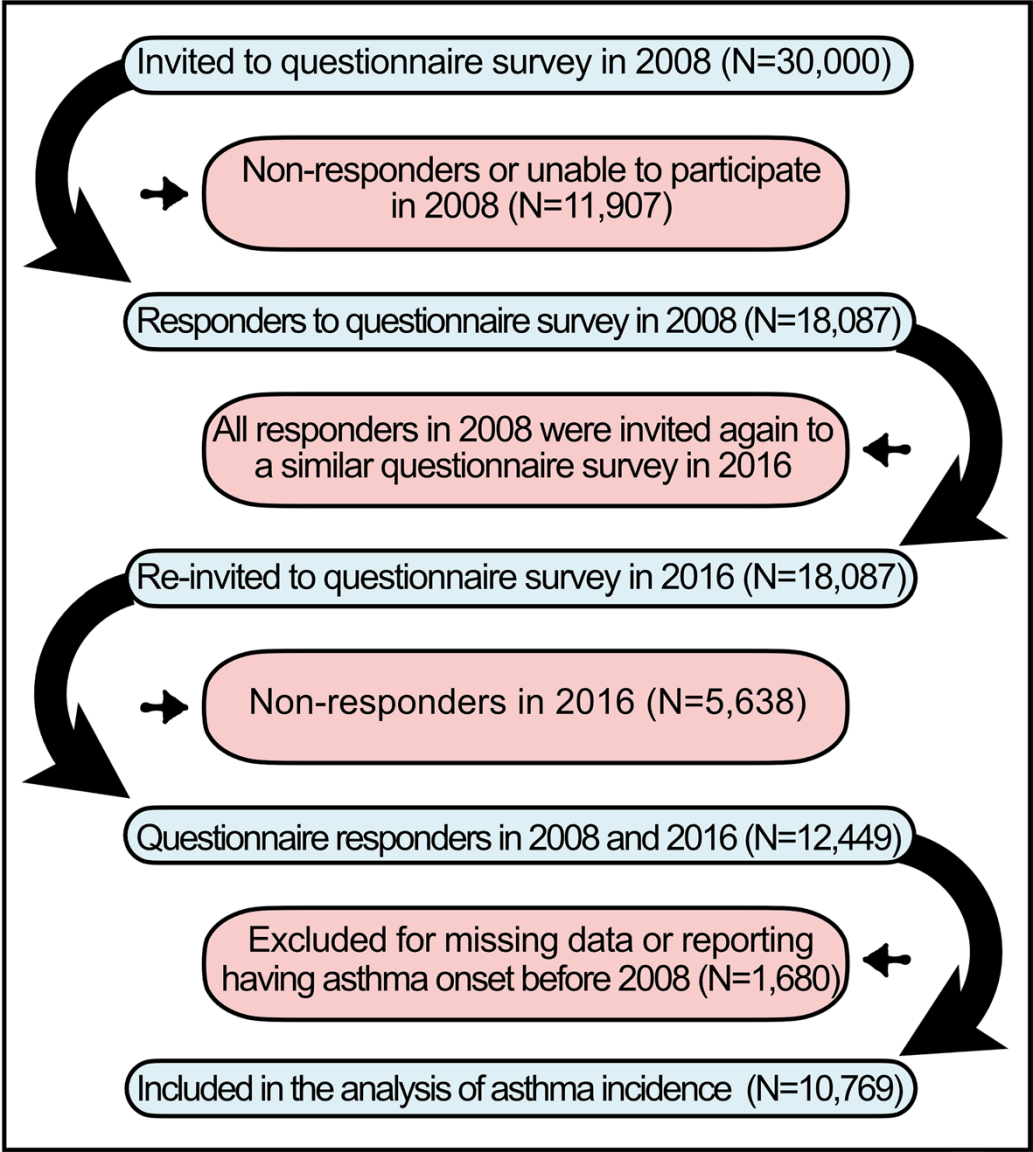
Flowchart of the West Sweden Asthma (WSAS) Study at baseline (2008) and follow-up in 2016.

The West Sweden asthma study (WSAS) population has been described previously in detail^27^. Shortly, WSAS is a longitudinal examination of adult individuals randomly selected from background population. Commencing in 2008, 30,000 subjects were chosen through the Swedish Population Register to take part in a postal questionnaire survey, in which 18,087 (60.3%) individuals took part in the study. Those who participated in 2008 were invited for a follow-up survey in 2016, of which 12,449 (69.1%) responded. After excluding those who reported having physician-diagnosed asthma in 2008 (*N* = 1,028), individuals with missing data (*N* = 390), and those having asthma onset before 2008 (*N* = 262), 10,769 participants have been included in our study (Fig. 1). Participants provided informed consent to the study protocol. The study was approved by the regional ethics board in Gothenburg, Sweden (The Swedish Ethical Review Authority). All methods were performed in accordance with the relevant guidelines and regulations.

### Data collection and questionnaire

At baseline (2008) and follow-up (2016), participants received a postal questionnaire that contained questions inquired about demographics, asthma, respiratory symptoms, allergies, environmental exposures and various risk factors.

### Statistical analysis

#### Variables definitions

Diagnosis of asthma was defined by a positive response to the question, “Have you been diagnosed with asthma by a physician?“. The age at asthma diagnosis was determined by asking, “At what age were you diagnosed with asthma?“. Body mass index (BMI) was calculated using self-reported height and weight. Obesity was defined as a BMI ≥ 30 kg/m², while overweight was defined as a BMI ≥ 25 and < 30 kg/m². Asthma characteristics were divided into four subtypes; symptomatic asthma, defined as asthma diagnosed during follow-up accompanied by at least one of asthma symptoms in the past 12 months (shortness of breath, wheezing, or a productive cough lasting over three months); medicated asthma, defined as asthma diagnosed during follow-up with reported use of asthma medications in the past 12 months; hospitalized asthma, defined as asthma diagnosed during follow-up with a history of hospitalization for asthma at any point during the follow-up period; and other asthma, defined as asthma diagnosed during follow-up without meeting the criteria for any of the other categories.

#### Asthma incidence

Asthma incidence rate (IR) was calculated after excluding individuals who reported having physician-diagnosed asthma in 2008. Incidence was measured within 8-year follow-up as new asthma diagnoses per 1000 person-years (py). Py were determined for each participant as the time from baseline (2008) until the age at diagnosis of asthma or otherwise set to the full follow-up period of 8 years for those who did not develop asthma. Incidence rates were calculated as:

$$\:Incidence\:Rate\:\left(IR\right)=\:\frac{Number\:of\:Events}{py\:at\:risk}\:\times\:\:1000$$

**Fig. 2:**
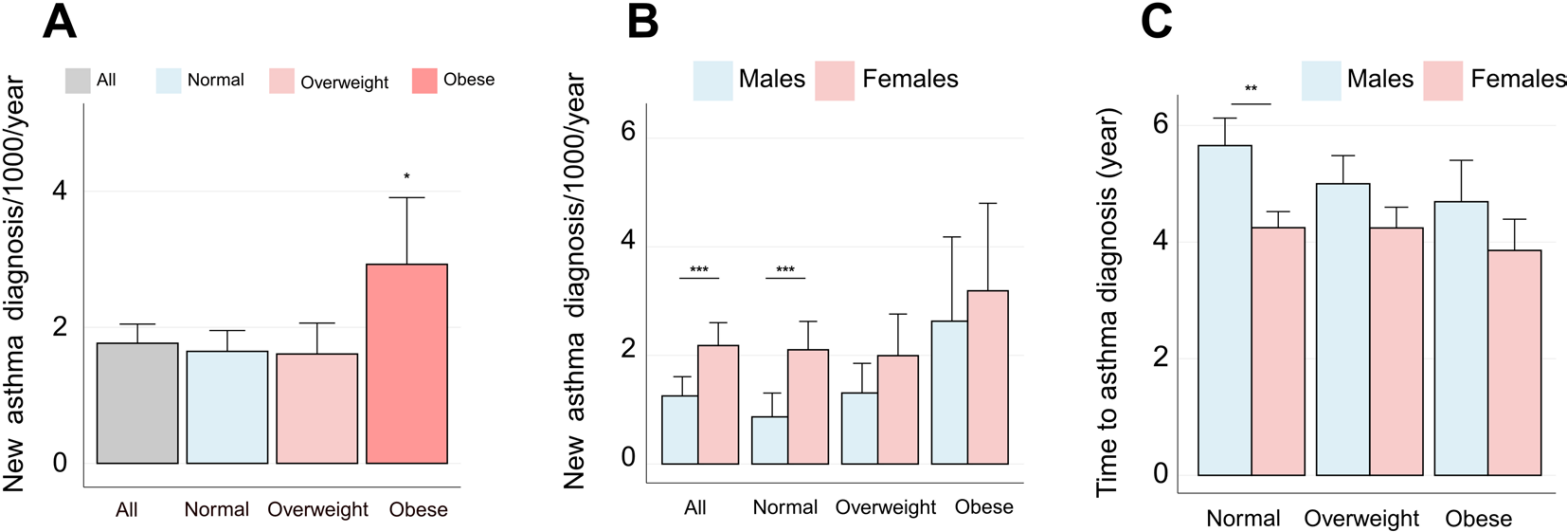
Asthma incidence and weight change by BMI Category. (**A**) Asthma incidence per 1,000 annually in the total study population. (**B**) Asthma incidence grouped by sex and obesity at baseline. (**C**) Average time (years) to develop asthma starting from baseline. Bars represent incidence or mean, and error bars indicate confidence intervals (CI). Statistical differences were assessed using Student’s *t-*test for mean comparisons and Wald test for difference in incidence rates. (*) symbol indicates *p*-value < 0.05, (**): *p*-value < 0.01, and (***): *p*-value < 0.001.

**Fig. 3:**
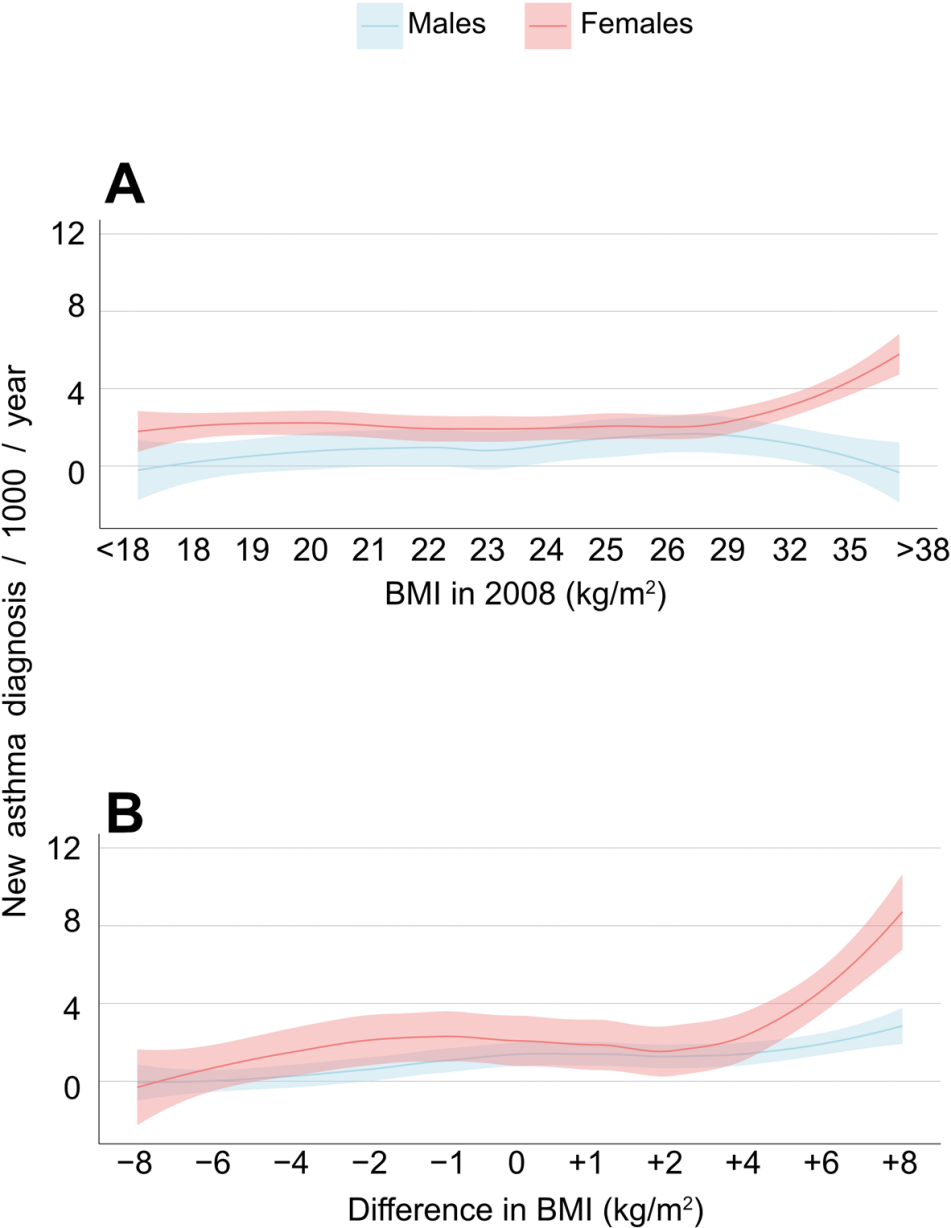
Asthma incidence rate (IR) in relation to BMI at baseline (**A**) and BMI (**B**) change over 8 years. (A) Asthma incidence based on participants’ BMI in 2008. (**B**) Asthma incidence based on BMI changes between 2008 and 2016. The curves show asthma incidence, and the shaded areas indicate the 95% confidence intervals. Note: incidence rate increased in females with > 32 BMI at baseline or > 4 BMI gain, but near-zero events in males limited statistical power to detect statistical differences.

**Fig. 4:**
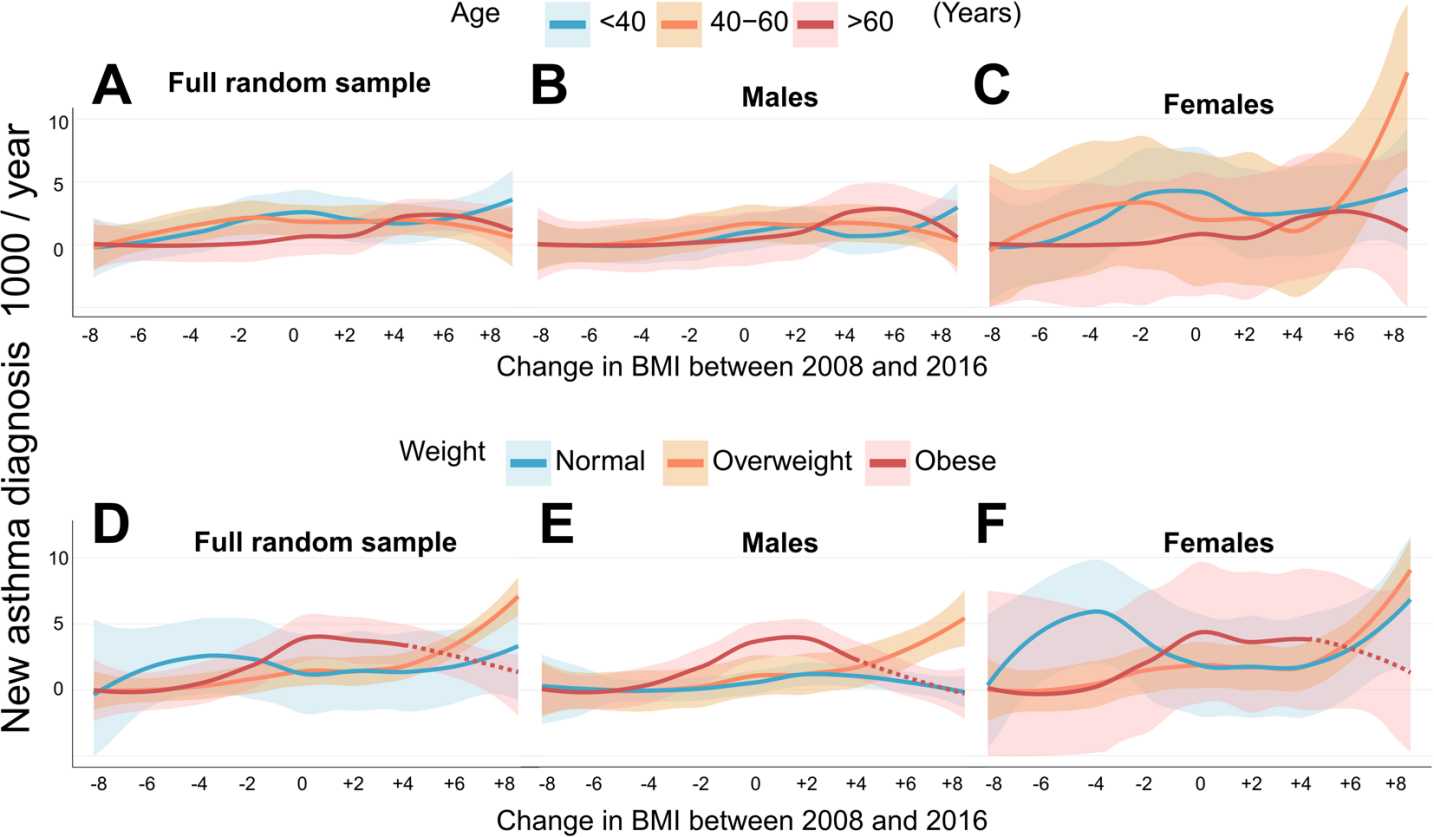
Asthma incidence based on BMI change from 2008 baseline to 2016 follow-up, broken down by age groups at 2008 [(**A**) full random sample (*N* = 10,769), (**B**) males (*n* = 4811), and (**C**) females (*n* = 5958)] and weight categories at 2008 [(**D**) full random sample, (**E**) men, and (**F**) women]. The shaded area represents the 95% confidence interval. Dotted lines show estimated curves where number of observations is too small to calculate exact asthma incidence.

Participants reporting asthma diagnoses beyond their current age or those who did not provide an age at diagnosis were excluded (*n* = 17, 4.0% of total asthma cases), but calculations of crude asthma incidence were adjusted for these cases after checking for their homogeneity as the following: $\:{IR}_{Adjusted}=\:{IR}_{crude}\:\times\:\:1.04$. This adjustment was applied to all crude asthma incidence results (Figs. 2, 3 and 4). To quantify uncertainty, 95% confidence intervals (CIs) were derived assuming a binomial distribution of events. The standard error (SE) of the incidence rate was computed as:

$$\:{SE\:=\:Z}_{0.975}\:\times\:\:\sqrt{\frac{r(1-r)}{py\:at\:risk}\:}\:\times\:\:1000\::\:r=\frac{Number\:of\:Events}{py\:at\:risk},{\:Z}_{0.975}=1.96$$

The 95% CIs were then constructed on the log scale to account for skewness of incidence rates:

$$\:Lower\:95\%\:Cl=\:{e}^{ln(IR-SE)}\:,\:Upper\:95\%\:Cl=\:{e}^{ln(IR+SE)}$$

Wald test for difference in rates was used to identify difference in IR (Figs. 2, 3 and 4) as follows:

$$\:z=\:\frac{{IR}_{1}-\:{IR}_{2}}{\sqrt{SE{{(IR}_{1})}^{2}+\:SE{{(IR}_{2})}^{2}\:}}\:;\:P\left(value\right)=\:P\left(Z>\:\left|z\right|\right):\:\:where\:Z\sim\:N\left(0,\:1\right)$$

#### Generalized linear models

Generalized linear models with a Poisson distribution and log link function were applied to estimate incidence rate ratios (IRRs) for incident asthma. In the primary models, asthma incidence was analyzed as a function of BMI change, with adjustment for sex and baseline BMI (continuous). We tested for effect modification by including interaction terms between BMI change and baseline age categorized into three groups (< 40, 40–59, ≥ 60 years). In a complementary set of models, asthma incidence was analyzed as a function of BMI change, with adjustment for sex and baseline age (continuous), while testing for interactions between BMI change and baseline BMI category (normal weight, overweight, obese). Model fit and the contribution of individual predictors were evaluated using likelihood ratio (ANOVA deviance) tests comparing nested models (Table S2). In the secondary models, we adjusted for sex and baseline BMI (continuous) to estimate IRRs stratified by age groups (Table 2).

**Fig. 5:**
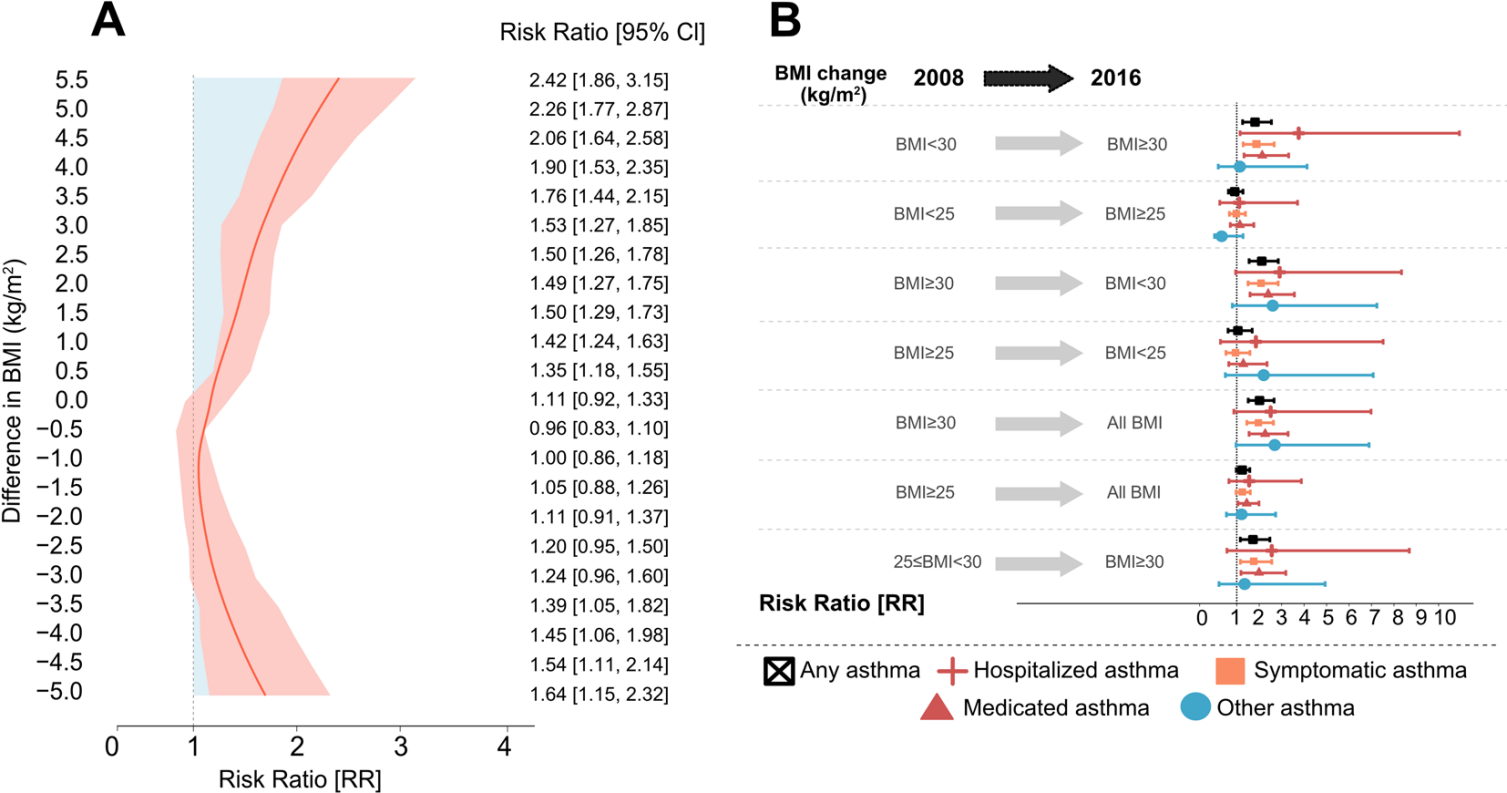
Risk ratio (RR) for asthma risk as outcome and BMI change as exposure over 8-year period. (**A**) BMI change (continuous). The curves show the cumulative RR of having asthma at follow-up. The red shaded areas represent confidence intervals (CIs), and the blue shaded areas highlight statistical significance. (**B**) BMI change (categorical). Dots represent age-adjusted RR, error bars indicate 95% confidence interval. Black color indicates any asthma cases (*n* = 419), red cross indicates hospitalized asthma cases (*n* = 36), orange color indicates symptomatic asthma cases (*n* = 380), red triangle represents medicated asthma cases (*n* = 247), and blue color indicates other asthma cases (*n* = 39). Both models adjusted for age and used participants with normal weight at baseline and change in BMI ≤ 1 kg/m^2^ at follow-up as a reference group.

Poisson regression with a log link was also applied to estimate risk ratios (RRs), treating BMI change as the main exposure. To address combinations of exposure and outcome with zero counts, a continuity correction of 0.1 was applied. RRs and their 95% confidence intervals (CIs) were obtained by exponentiating the model coefficients. In the full sample, models were adjusted for baseline age (continuous) to estimate RRs for any asthma and its subtypes (Fig. 5).

**Fig. 6:**
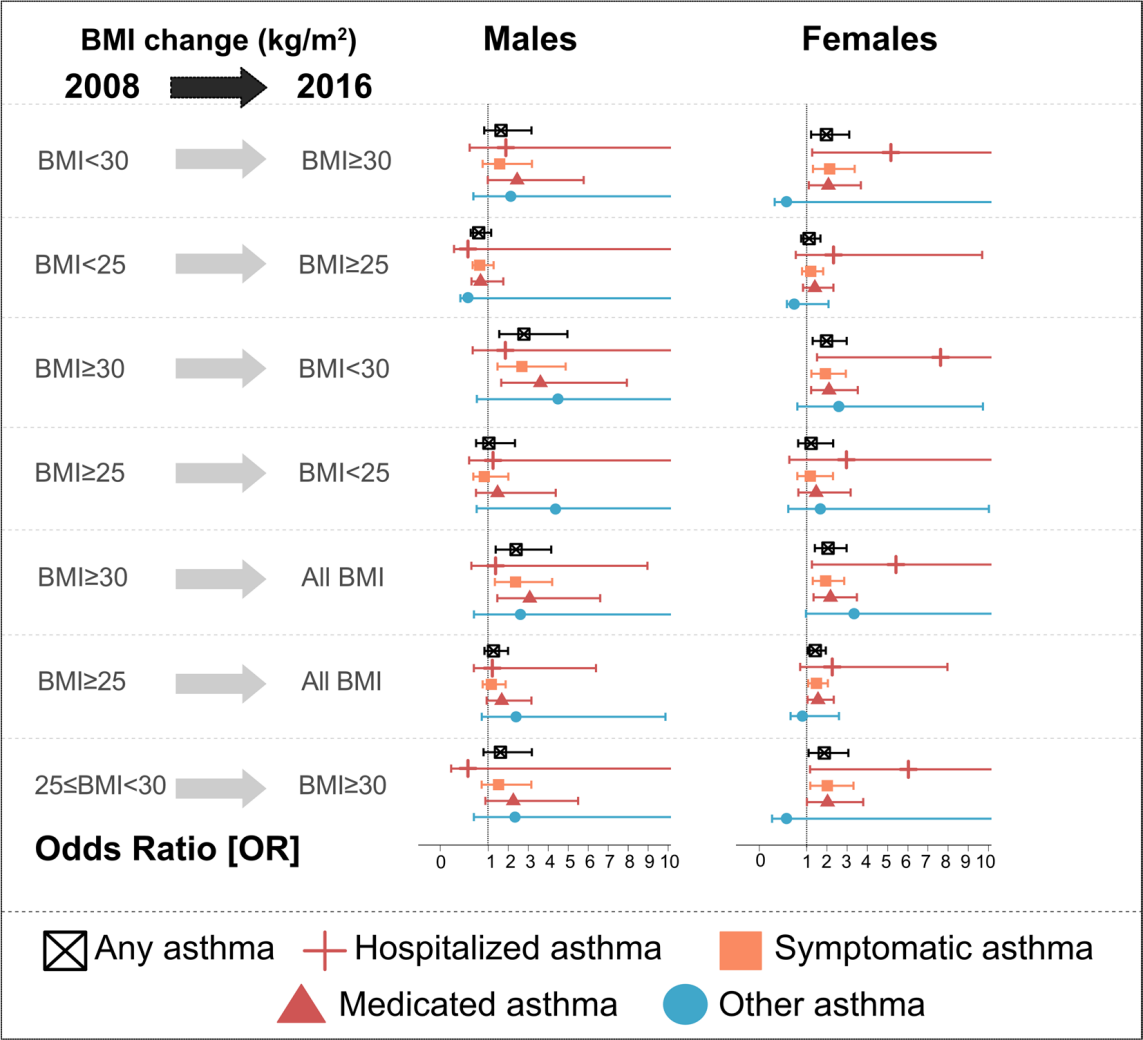
Odds ratio (OR) for any asthma risk and asthma subtypes risk as outcome and BMI change (categorical) as exposure over 8-year period divided by sex. ORs were calculated separately for males and females cohorts using age-adjusted binary logistic regression models. Dots represent age-adjusted odd ratio, error bars indicate 95% confidence interval. Black color indicates any asthma cases, red cross indicates hospitalized asthma cases, orange color indicates symptomatic asthma cases, red triangle represents medicated asthma cases, and blue color indicates other asthma cases. Males or females with normal weight at baseline and change in BMI ≤ 1 kg/m^2^ at follow-up were used as a reference group.

For sex-stratified analyses of asthma subtype risk, some groups had too few cases to calculate robust RRs using Poisson regression; in these cases, age-adjusted binary logistic regression models were used to estimate odds ratios (ORs) (Fig. 6), which provide reliable approximations of RRs for rare outcomes^28^.

Statistical significance was considered at a threshold of *p* < 0.05. However, interaction terms are typically considered exploratory and may be interpreted at a more lenient significance threshold^29,30^. Therefore, *p* value of *p* < 0.1 were considered significant for interaction terms. Statistical analyses were conducted utilizing RStudio software (Version-2023.12.1 Build-402, Posit-Software).

### Research in context

#### Evidence before this study

Asthma and obesity are common public health concerns. Obesity is linked to an increased risk of multiple health conditions, including cardiovascular disease, type 2 diabetes, and respiratory disorders such as asthma. When we searched Medline and Scopus without time or language restrictions for studies related to obesity, weight change, and asthma risk or incidence, we found that several cross-sectional and cohort studies have found that obesity increases both the incidence and severity of asthma. Obese individuals with asthma often experience worse symptoms, more frequent exacerbations, and poor disease control, requiring higher doses of inhaled corticosteroids. However, we found no study that examined these outcomes in a general population setting. While many studies have identified a dose-dependent relationship between higher BMI and asthma risk, the specific BMI threshold at which risk increases remains unclear. All found existing research has focused on single BMI measurements rather than weight changes over time. Only four studies have examined whether weight gain or loss influences asthma risk, and these were often limited by participant age range or sex, making their findings less generalizable. Additionally, no studies have explored how weight changes affect asthma risk across different age groups and weight categories. These literature research outcomes were in parallel with a recent systemic review report as well (Parasuaraman et al. 2023).

#### Added value of this study

This study provides new insights into the relationship between obesity, weight change, and asthma risk using data from an 8-year follow-up of a population-representative adult cohort. Unlike previous research, it accounts for weight fluctuations over time rather than relying on a single BMI measurement. It also examines asthma risk across different age groups and baseline weight categories, offering a more comprehensive view of how weight changes influence disease development. By considering factors such as age, sex, and initial BMI, this study helps clarify whether asthma risk is driven by obesity itself or by weight gain over time.

#### Implications of all the available evidence

The findings suggest that both obesity and weight changes play a role in asthma development, with potential implications for disease prevention and management. Public health strategies should focus not only on reducing obesity rates but also on promoting weight stability, particularly in high-risk groups. Future research should explore the underlying mechanisms linking weight changes to asthma risk and determine whether interventions targeting weight control can reduce asthma incidence. Additionally, studies should assess how age and baseline BMI interact with weight changes to influence long-term respiratory health.

## Results

Of those without physician-diagnosed asthma in 2008, 430 (3.9%) reported asthma in 2016. Seventeen (4.0%) had missing data on age of onset and were excluded, but incidence rates were adjusted for them. Another 262 (60.1%) reported asthma onset before 2008 and were excluded without adjustment. The remaining 151 participants developed physician-diagnosed asthma with onset between 2008 and 2016. They were generally younger, more often women, and had similar BMI and smoking status compared with those without asthma in 2016 (Table 1). For study population and weight change characteristics based on BMI categories, see Table 1S and Fig. 1S.

**Table 1:** Characteristics of participants at baseline in 2008 (*N* = 10,769).

	All participants (*N* = 10,769)	No asthma at 2016 (*N* = 10,618, 98.6%)	Asthma at 2016 (*N* = 151, 1.4%)
Sex (male)	4,811 (44.7%)	4,763 (44.9%)	48 (31.8%)
Age (y)	48.1 (15.4)	48.1 (15.4)	43.9 (13.6)
Age at asthma onset (y)	47.7 (14.1)	NA	47.7 (14.1)
Time to asthma (y)	3.8 (2.2)	NA	3.8 (2.2)
BMI	25.1 (4.1)	25.1 (4.1)	25.5 (4.4)
Normal weight (BMI < 25 kg/m^2^)	5,889 (54.7%)	5,812 (54.7%)	77 (51.0%)
Overweight (30 > BMI ≥ 25 kg/m^2^)	3,756 (34.9%)	3,708 (34.9%)	48 (31.8%)
Obese (BMI ≥ 30 kg/m^2^)	1,124 (10.4%)	1,098 (10.3%)	26 (17.2%)
Current smoker	1,408 (13.5%)	1,384 (13.5%)	24 (16.3%)
Ex-smoker	2,735 (29.2%)	2,701 (29.3%)	34 (25.2%)
Education (> 12 years)	4,447 (41.8%)	4,381 (41.7%)	66 (44.3%)

Data was presented as n (%) or mean (SD).

BMI = Body Mass Index, NA = non-applicable.

### Asthma incidence

Asthma incidence was 1.8 per 1,000-py in the entire study population. Normal and overweight individuals had similar asthma rates, but incidence was significantly higher in obese participants in the whole cohort (Fig. 2A). Additionally, asthma incidence was significantly higher in females than in males, both in the full sample and across normal weight category, and insignificantly higher in overweight and obese participants (Fig. 2B). Females developed asthma in shorter time than males in the normal weight category. However, this difference disappeared in overweight and obese categories (Fig. 2C).

To better understand how obesity affects the risk of developing asthma, we calculated asthma incidence based on participants’ BMI in 2008 (Fig. 3A). Asthma incidence was consistently higher in females with a BMI < 29 (kg/m^2^) than males with the same BMI. For females with a BMI > 29 (kg/m^2^), the incidence of asthma increased sharply. However, in males, asthma incidence rose more gradually by increasing BMI, where the rates in males were close to those in females at a BMI of 29 (kg/m^2^), but oppose to women, the incidence in males declined with BMI > 29 (kg/m^2^) (Fig. 3A). However, because events were absent in males for those with BMI > 32 (kg/m^2^), our study had limited power to detect statistical differences between males and females.

Furthermore, we wanted to assess the impact of BMI change on asthma risk. Thus, asthma incidence was calculated based on participants’ BMI change between 2008 and 2016 (Fig. 3B). Asthma incidence in females who gained ≥ 4 BMI (kg/m²) rose more sharply than in men. However, because events were nearly absent in males for those who gained ≥ 4 BMI (kg/m²), our study had limited power to detect statistical differences between males and females.

To better understand how weight changes affect asthma incidence based on age and obesity, we stratified the above analysis by baseline age (Fig. 4A–C) and BMI categories (Fig. 4D–F). Asthma incidence was highest among those in younger ages (≤ 40 years) and middle age (40–60 years) than older participants (> 60 years) (Fig. 4A). In men, asthma incidence was highest in those who gained BMI units, with no clear age-related pattern (Fig. 4B). In women, those in middle age (40–60 years) and younger females (≤ 40 years) had the highest asthma incidence, regardless of BMI change, compared to older females (> 60 years). Additionally, asthma rates among middle-aged females increased significantly when increase of BMI exceeded 4 (kg/m^2^) (Fig. 4C). In our regression model, we categorized baseline age into three groups (< 40, 40–60, ≥ 60 years) and tested the interaction between BMI change and age groups. The model adjusted for age groups, sex, and baseline BMI (continuous). Both BMI change (*p* = 0.007) and age groups (*p* < 0.001) were independently associated with asthma incidence. Their interaction also showed suggestive evidence of association (*p* = 0.077) (Table 2S). Because a significant interaction was observed between BMI change and age groups, analyses were stratified by age groups (< 40, 40–59, ≥ 60 years). In adults < 40 years, BMI change was not associated with asthma incidence, but females had higher incidence than males. Among those aged 40–59 years, higher BMI at baseline was significantly associated with increased asthma risk, while BMI change remained non-significant. In adults ≥ 60 years, BMI change was associated with higher asthma incidence, while sex and BMI at baseline were not significant predictors (Table 2).

**Table 2:** Stratified Poisson regression analysis of predictors of incident asthma, by age group.

Age groups	Predictor	IRR (95% CI)	*p*-value
< 40 (year)	BMI change (for each 2 unit)	1.07 (0.87–1.31)	0.540
	Sex (female)	2.15 (1.21–4.09)	**0.013**
	BMI baseline (for each 1 unit)	1.15 (0.73–1.70)	0.520
	BMI change (for each 2 unit)		
40–60 (year)	Sex (female)	1.15 (0.92–1.42)	0.220
	BMI baseline (for each 1 unit)	1.72 (1.04–2.95)	**0.040**
		1.60 (1.15–2.22)	**0.005**
	BMI change (for each 2 unit)		
≥ 60 (year)	Sex (female)	1.66 (1.12–2.40)	**0.009**
	BMI baseline (for each 1 unit)	1.29 (0.52–3.34)	0.590
		1.57 (0.82–2.96)	0.170

IRR: incidence rate ratios, Cl: confidence Interval.

Asthma incidence tended to increase with weight gain in overweight participants and, to a lesser extent, in those with normal weight. Obese participants had the highest asthma incidence regardless of weight stability or gain. However, asthma incidence decreased in obese participants who lost weight. There were too few obese individuals who gained ≥ 4 BMI units to calculate asthma incidence in this group (Fig. 4D). In men, asthma incidence rose with weight gain in overweight males but not in those with normal weight (Fig. 4E). Asthma incidence was highest in obese females without weight changes and spiked sharply in normal and overweight females who gained ≥ 4 BMI (kg/m²) by 2016. Obese females followed a similar trend to obese men. However, unlike males, females with normal weight only (not all weight categories) at baseline who lost weight experienced a rise in asthma incidence (Fig. 4F). We categorized baseline BMI into three groups (normal, overweight, obese) and tested the interaction between BMI change and baseline-BMI groups adjusting for sex and age (continuous). BMI changes were significantly associated (*p* = 0.007) and baseline-BMI groups were almost significantly associated (*p* = 0.058) with asthma incidence independently. Their interaction, however, showed no significant association (*p* = 0.723) (Table 2S). We found no difference in asthma incidence rates in participants with stable BMI category with or without weight change (Fig. 2S).

### Obesity and weight change in relation to the risk of asthma

After observing a link between weight gain and asthma incidence, we aimed to quantify the risk and identify the BMI change threshold where the risk becomes significant. Using an unadjusted generalized linear model with a log link function for binomial data, we calculated risk ratios for asthma based on weight change as the exposure. The risk of having asthma was significantly higher in those who gained ≥ 0.5 BMI (kg/m²) (Fig. 5A) from baseline and increased by increasing BMI change between 2008 and 2016. Asthma risk was also significantly higher in those who lost ≥ 3.5 BMI (kg/m²).

Furthermore, we aimed to assess how BMI change influences the risk of various asthma characteristics, accounting for age as a potential confounding factor. We used an age-adjusted generalized linear model with a log link function for binomial data to calculate the RR of having asthma based on BMI changes, accounting for baseline BMI. Obesity at baseline was linked to a significantly higher asthma risk, regardless of weight change. The risk was also elevated for participants who became obese or transitioned from overweight to obesity by 2016, compared to those with normal weight at both time points and minimal BMI change [≤ 1 BMI (kg/m²)] (Fig. 5B).

Unlike other asthma, the risk of medicated or symptomatic asthma was higher in individuals with obesity at baseline, those who lost obesity, or those who became obese or progressed from overweight to obesity during follow-up. Notably, medicated asthma risk was also elevated in those who were overweight at baseline. Lastly, the risk of hospitalized asthma increased significantly only in individuals who became obese by follow-up (Fig. 5B).

Both in males and females, medicated and symptomatic asthma was higher in individuals with obesity at baseline. However, unlike in men, hospitalized asthma risk was significant among obese females at baseline or those who changed obesity status during follow-up, even if they only transitioned from overweight to obesity (Fig. 6).

## Discussion

In our study, asthma incidence was significantly higher in obese participants at baseline than in those with normal weight. Females had higher asthma incidence than men, both in the full sample and within normal BMI category. Among Females with BMI > 29 (kg/m^2^) at baseline or those who gained ≥ 4 BMI (kg/m²) over eight years, asthma incidence increased numerically compared to males with similar BMI or weight gain. Asthma incidence was highest among females who gained weight in middle age (40–60 years), followed by younger (≤ 40 years) and older participants (> 60 years). Asthma incidence also rose among obese males and females who did not lose weight, and overweight who gained weight. However, asthma incidence spiked in normal weight females who changed ≥ 4 BMI units during the 8-year period. Asthma risk was significantly higher among those who gained weight, even with an increase of ≥ 0.5 BMI (kg/m²) from baseline, with risk rising as weight gain increased for 8 years. Finally, obesity was associated with increased risk to asthma being more symptomatic and medicated and requiring hospitalization, especially among females. These outcomes highlight that effective weight management is crucial in clinical practice to prevent asthma and to facilitate asthma management.

Our study found that asthma incidence in the full cohort was higher among obese participants compared to those with normal weight, consistent with previous research showing a link between obesity and asthma incidence^11–19^. However, no increase in asthma incidence was observed among overweight participants compared to normal-weight individuals. While some studies have reported an increased risk^12–15^, discrepancies may stem from their limited sample size or narrow age range, not reflecting the full adult lifespan. Our findings align with studies that included a broader, more representative age range and found no increased asthma incidence among overweight participants^11,20^. Furthermore, asthma incidence was significantly higher in females than in males in the whole cohort, consistent with previous studies showing that adult-onset asthma is predominant in females^9,31–34^. The difference in asthma incidence in the full cohort between males and females disappeared in overweight and obese participants, suggesting that obesity may affect or override the influence of sex hormones on asthma^35^.

Many cohort studies have examined asthma incidence based on participants’ BMI at baseline^11–19^. However, most focused on obesity categories only and did not consider BMI as a continuous variable. Our study is the first to evaluate the continuous nature of BMI on asthma incidence. Hjellvik et al.^18^ found that asthma incidence increased in men with a baseline BMI ≥ 25 (kg/m^2^) and rose sharply in women with the same BMI. Similarly, Camargo et al.^14^ reported a sharp increase in asthma risk among women with a BMI ≥ 25 (kg/m^2^) at baseline. In our study, asthma incidence rose among females with a baseline BMI ≥ 30 (kg/m^2^), but not in males. The difference between our findings and previous studies may be due to the different length of follow-up period between the studies. However, both studies highlight the importance of investigating the sex hormones roles in asthma development, especially that sex hormones have been linked to worse symptoms in asthma patients^36,37^.

The study design does not allow for causal conclusions, leaving potential confounding factors such as ethnicity, physical activity, diet, or comorbidities unaddressed. For instance, we observed that asthma incidence increased among normal-weight females who lost significant weight during follow-up. Additionally, asthma risk was higher in those who were obese at baseline but no longer obese at follow-up. Furthermore, the risk of asthma risk with weight change has shown U-shaped relationship with those losing ≥ 3.5 BMI (kg/m^2^) had higher asthma risk. This may be due to comorbidities that developed during follow-up, leading to misclassification of asthma diagnoses. Alternatively, obesity might have caused asthma, with weight loss occurring after the diagnosis, or the inflammatory patterns linked to obesity could persist during weight loss. Adipose tissue in obesity secretes pro-inflammatory mediators—including IL-6 and leptin—that contribute to systemic and airway inflammation. For instance, IL-6 is elevated in obesity and asthma and has been linked to worse asthma outcome and increased medication use^38^. Leptin, an adipokine, promotes airway inflammation through activation of immune cells, including eosinophils and lung fibroblasts, and may drive airway remodeling^39^. These mechanisms may still have influenced the risk of asthma after weight loss. In addition, it is worthy to note that obesity can itself cause respiratory symptoms such as breathlessness, even without active asthma. This is particularly common in females and may contribute to a higher rate of false asthma diagnoses in this group^40^.

To our knowledge, no study has assessed the impact of weight change on asthma incidence by stratifying participants based on age, and weight categories at baseline. Brumpton et al.^17^ reported that obesity is linked to higher odd ratios only in Norwegian adults aged ≥ 40 years. Similarly, Coogan et al.^19^ found that overweight and obesity were associated with higher relative risk in women aged ≥ 40 years, while obesity alone was significant in women < 40 years. In our regression models, baseline BMI and BMI change were not associated with asthma incidence in younger participants. In middle-aged participants, higher baseline BMI was linked to increased asthma risk. In older participants, BMI change was associated with higher asthma incidence. Furthermore, we observed that individuals < 40 years and those aged 40–60 years had higher asthma incidence rates than those > 60 years, even without weight change, particularly among women. Asthma incidence increased in middle-aged females compared to older females. Additionally, this group of females had the highest asthma incidence rate across all age groups when gained ≥ 4 BMI (kg/m^2^). Therefore, our study shows that women who are non-obese and gain notable weight or are obese in middle age are the most likely to develop asthma compared to other age, sex, and weight groups. This is particularly important because middle-aged women are the most likely to gain weight during their lifetime^41^. In addition, sex hormone levels drop during menopause, creating different fluctuation patterns that may worsen asthma incidence and symptoms in females^37^.

The risk of developing asthma in adulthood was significantly higher for individuals with substantial weight gain, as shown in previous studies^14,19,23^. Among white or black American women, gaining ≥ 10 kg/4-year period or ≥ 15 kg/10-year period, respectively, increased the RR for asthma^14,19^. In American adults over 40 years, a 20 kg weight gain also increased the risk^23^. In our study, we found that gaining ≥ 0.5 BMI (kg/m²) from baseline over an eight-year follow-up significantly increased the risk of developing asthma, with the risk rising further with greater BMI gain. These differences in thresholds may be explained by variations in study design. First, the follow-up periods and reference groups differed between studies. For instance, previous studies used individuals with weight change (from − 2 to + 2.5 kg) as reference groups^14,19,23^, whereas we used participants with normal weight and less than 1 BMI unit of change. It is worthy to note that using individuals only with minimal or no weight change as the reference group, without considering their baseline BMI category, can hide distinctions between overweight or obese participants and normal-weight, weight-stable individuals. Second, previous studies often had narrow inclusion criteria, such as focusing solely on women or specific age ranges, whereas our study included a population-representative sample, enhancing generalizability. These methodological differences likely contribute to the variability in weight gain thresholds reported across studies.

For the first time, our study investigates the link between obesity and the burden of asthma in population-representative settings. We found that obese individuals face a higher risk of developing symptomatic asthma, unlike those with other asthma, requiring medication and hospitalization compared to those with normal weight, especially among women. Consistent with previous studies, Hasegawa et al.^42^ reported that obese individuals have a higher risk of hospitalization after ER visits than those with normal weight. Obesity has also been linked to greater disease severity, hospitalizations, higher symptom burden, and higher medication use and doses^43–45^. However, unlike earlier research, our study is one of the first to examine these outcomes in a community-based setting. Our results also support the clinical data showing that obese women are at greater risk of developing incident asthma that is related to hospitalizations which is less obvious in obese men. These findings suggest that obesity could affect men and women differently in clinical outcomes: both experience more symptoms, but women may respond less effectively to treatment.

Our study has several strengths that make its findings robust and broadly applicable. First, we analyzed a large, population-representative sample drawn from West Sweden, ensuring diverse age, sex, and weight groups, which enhances the generalizability of our results. The eight-year follow-up period allowed us to capture long-term trends in weight change and asthma incidence. Unlike many previous studies, we considered BMI as a continuous variable and assessed the impact of weight gain across different age, sex, and baseline weight categories. This approach provided insights into the interaction between these factors and asthma risk, particularly in middle-aged women who significantly gained weight. However, there are limitations to consider. First, asthma diagnosis and weight were self-reported, which may lead to misclassification bias and affect both precision and validity of our estimates. Such information bias is well-documented, particularly when outcome measurement relies on recall. The lack of objective measures for asthma diagnosis, such as clinical assessments or spirometry, limits the precision of our findings. However, in large population-level epidemiological studies this method is commonly used as it is challenging to confirm every diagnosis in detail with lung function testing in non-medicated patients^34,46^. For example, age at diagnosis has been very reliable against a lung function confirmed diagnosis age^46^. Second, while we adjusted for key demographic factors, we did not include other potential confounders such as comorbid conditions (e.g., COPD, gastroesophageal reflux), medication use, or inflammatory biomarkers like eosinophil counts. These factors may independently influence asthma risk, and their omission may bias our results. Despite these limitations, our study provides valuable insights into the role of obesity and weight change in asthma incidence, emphasizing the need for effective weight management particularly for middle-aged women (40–60 years) who are most prone to weight gain^41^, to reduce the risk of asthma.

Our study underscores the critical role of obesity and weight gain in the development of asthma and asthma management. Clinically, our findings highlight the necessity for health care to integrate weight management strategies as a key component of asthma prevention and care, particularly for women in middle age who are overweight or obese. Asthma risk significantly rises with even modest weight gain, emphasizing the importance of early interventions to promote healthy weight maintenance. Thus, public health efforts should focus on addressing obesity as a modifiable risk factor for asthma. Furthermore, obesity may also exacerbate symptoms, reduce treatment efficacy, and worsen outcomes^42,44,45^, making weight control an essential factor in asthma management plans, especially in severe asthma clinics.

In summary, our study highlights a significant association between obesity, weight gain, and the incidence of asthma, particularly in females who gain weight during middle age. Obese individuals at baseline had a higher risk of developing asthma compared to those with normal weight. Weight gain, even as modest as 0.5 BMI (kg/m^2^), significantly increased asthma risk, with the highest incidence observed in normal to overweight females who gained ≥ 4 BMI (kg/m^2^) over an 8-year period. These findings underscore the importance of weight management in preventing asthma, particularly in middle-aged women.

## Supplementary Information

Below is the link to the electronic supplementary material.

Supplementary Material 1

## Data Availability

Data is considered a sensitive data and it is protected by the European Union General Data Protection Regulation (GDPR) law regulation. For requests regarding the data, please contact the corresponding author.

## Abbreviations

BMI: Body mass index
Cls: Confidence intervals
ECRHS: The European community respiratory health survey
GA2LEN: The global allergy and asthma European network
Kg: Kilogram
OLIN: Obstructive lung diseases in northern Sweden
OR: Odds ratio
RR: Risk ratio/relative risk
WSAS: West Sweden asthma study
