# Supplementary material for: Obesity and weight change during eight years in relation to asthma incidence
Source: Sci Rep. 2025 Oct 7;15:35017. doi: 10.1038/s41598-025-20657-8 (PMC12504545; doi:10.1038/s41598-025-20657-8)
Supplement: Supplementary file 1 — Supplementary Material 1 [file 41598_2025_20657_MOESM1_ESM.docx]

Obesity and weight change during eight years in relation to asthma incidence

*Reshed Abohalaka^1^, Selin Ercan^1^, Pinja Ilmarinen^2,3^, Helena Backman^4^, Linda Ekerljung^1,5^, Madeleine Rådinger^1^, Bright I. Nwaru^1,6^, Hannu Kankaanranta^1,2,3^*

^1^Krefting Research Centre, Department of Internal Medicine and Clinical Nutrition, Institute of Medicine, Sahlgrenska Academy, University of Gothenburg, Gothenburg, Sweden
^2^Department of Respiratory Medicine, Seinäjoki Central Hospital, Seinäjoki, Finland
^3^Faculty of Medicine and Health Technology, Tampere University, Tampere, Finland

^4^Department of Public Health and Clinical Medicine, Umeå University, Umeå, Sweden

^5^Department of Internal Medicine/Respiratory Medicine and Allergology, The Sahlgrenska University, Gothenburg, Sweden
^6^Wallenberg Centre for Molecular and Translational Medicine, University of Gothenburg, Sweden

Corresponding author: Reshed Abohalaka, MSc

Krefting Research Centre

Department of Internal Medicine and Clinical Nutrition

Institute of Medicine

Sahlgrenska Academy

Medicinaregatan 1F, Box 424, University of Gothenburg, 405 30 Gothenburg, Sweden

Tel: +46 31 786 67 12

e-mail: [reshed.abohalaka@gu.se](mailto:reshed.abohalaka@gu.se)

**Methods:**

**Study area and population**

The West Sweden Asthma Study (WSAS) population has been described previously in detail ^1^. Shortly, WSAS is a longitudinal examination of 16- to 75-year-old individuals randomly selected from background population. Commencing in 2008, 30,000 subjects within the specified age range were chosen through the Swedish Population Register to take part in a postal questionnaire survey. The selection process was stratified based on age and gender to ensure a representative reflection of the West Gothia population in Sweden. After excluding those untraceable (n=782), 18,087 (60.3%) individuals took part in the study. Those who participated in 2008 were invited for a follow-up survey in 2016, of which 12,449 (69.1%) responded. After excluding those who reported having physician-diagnosed asthma in 2008 (N = 1028), individuals with missing data (N = 390), and those having asthma onset before 2008 (N = 262), 10,769 participants have been included in our study (**Figure 1**). Participants consented to a study protocol approved by the regional ethics board in Gothenburg, Sweden.

**Data Collection and Questionnaire**

At baseline, participants received a postal questionnaire that contained questions that have been previously used in the Obstructive Lung Diseases in Northern Sweden (OLIN) ^2^, the Global Allergy and Asthma European Network (GA2LEN) studies ^3^, the FinEsS studies in Finland, Estonia and Sweden ^4^, and the European Community Respiratory Health Survey (ECRHS) ^5^. The self-administered questionnaire inquired about demographics, asthma, respiratory symptoms, allergies, environmental exposures and various risk factors. The applicable sections of the questionnaire were translated into Swedish before use in the study.

**Table 1S:** Baseline characteristics of participants in 2008 (N = 10,769)

| **No asthma in 2016**  (n =10,618, 98.6%) | | | | **Asthma in 2016**  (n =151, 1.4%) | | | |
| --- | --- | --- | --- | --- | --- | --- | --- |
|  | **Normal** | **Overweight** | **Obese** |  | **Normal** | **Overweight** | **Obese** |
| n (%) | 5812 (54.7%) | 3708  (34.9%) | 1098  (10.3%) |  | 77 (50.1%) | 48  (31.8%) | 26  (17.2%) |
| Sex (Male) | 2,154 (37.1%) | 2,092 (56.4%) | 517 (47.1%) |  | 15 (19.5%) | 22 (45.8%) | 11 (42.3%) |
| Age (y) | 44.6 (15.9) | 52.4 (13.7) | 52.5 (13.5) |  | 38.7 (13.5) | 49.3 (11.7) | 48.9 (11.1) |
| Age at asthma onset (y) | Non-applicable | | |  | 42.4 (14.2) | 53.4 (12.0) | 52.8 (11.2) |
| BMI | 22.3 (1.8) | 27 (1.4) | 33.3 (4.0) |  | 22.1 (1.8) | 27 (1.5) | 32.7 (3.0) |
| Current smoker | 737 (13%) | 471 (13.2%) | 176 (16.5%) |  | 14 (18.4%) | 8 (17.4%) | 2 (8.0%) |
| Ex-smoker | 1,201 (23.8%) | 1,124 (34.7%) | 376 (39.5%) |  | 14 (21.5%) | 10 (22.2%) | 10 (40.0%) |
| Education (>12 years) | 2,776 (48.4%) | 1,293 (35.2%) | 312 (28.7%) |  | 39 (50.6%) | 16 (34.0%) | 11 (44.0%) |
|  | **Change in BMI (units) at follow-up** | | | | | | |
|  | **Lost ≥2.5** | **Stable** | **Gained ≥2.5** |  | **Lost ≥2.5** | **Stable** | **Gained ≥2.5** |
| n (%) | 577 (5.3%) | 8,404 (79.2%) | 1,637 (15.4%) |  | 5 (3.3%) | 115 (76.2%) | 31 (20.5%) |
| Sex (Male) | 250 (43.3%) | 3,884 (46.2%) | 629 (38.4%) |  | 1 (20%) | 41 (35.7%) | 6 (19.4%) |
| Age (y) | 53.0 (15.3) | 48.8 (15.1) | 42.8 (16.0) |  | 37.2 (13.8) | 44.7 (13.6) | 41.7 (13.4) |
| Age at asthma onset (y) | Non-applicable | | |  | 41.2 (15.4) | 48.4 (14.0) | 41.2 (15.4) |
| BMI | 30.0 (6.5) | 24.7 (3.6) | 25.1 (4.2) |  | 28.9 (5.2) | 25.4 (4.4) | 25.3 (4.4) |
| Normal weight (BMI<25 kg/m^2^) | 99 (17.2%) | 4,822 (57.4%) | 891 (54.4%) |  | 2 (40.0%) | 61 (53.0%) | 14 (45.2%) |
| Overweight (30>BMI≥25 kg/m^2^) | 254 (44%) | 2,918 (34.7%) | 536 (32.7%) |  | 1 (20.0%) | 33 (28.7%) | 14 (45.2%) |
| Obese (BMI≥30 kg/m^2^) | 224 (38.8%) | 664 (7.9%) | 210 (12.8%) |  | 2 (40.0%) | 21 (18.3%) | 3 (9.7%) |
| Current smoker | 91 (16.5%) | 1,002 (12.3%) | 291 (18.4%) |  | 0 (0%) | 16 (14.3%) | 8 (26.7%) |
| Ex-smoker | 195 (38.5%) | 2,164 (29.4%) | 342 (25.1%) |  | 0 (0%) | 27 (26.5%) | 7 (25.0%) |
| Education (>12 years) | 192 (33.5%) | 3,583 (43.1%) | 606 (37.4%) |  | 4 (80.0%) | 52 (45.6%) | 10 (33.3%) |

Data was presented as n (%) or mean (SD). BMI = Body Mass Index.

**Table 2S:** Results of likelihood ratio tests from Poisson regression models for incident asthma.

| Model 1* |  |  |  |  |
| --- | --- | --- | --- | --- |
| Predictor | Deviance | Residual df | Residual deviance | p-value |
| BMI change (for each 2 unit) | 7.23 | 10,756 | 1433.9 | **0.007** |
| Age groups | 11.71 | 10,755 | 1422.2 | **<0.001** |
| Sex (Female) | 8.57 | 10,754 | 1413.6 | **0.003** |
| BMI baseline (for each 1 unit) | 8.98 | 10,753 | 1404.6 | **0.003** |
| BMI change vs age groups | 3.13 | 10,752 | 1401.5 | **0.077** |
|  |  |  |  |  |
| Model 2** |  |  |  |  |
| Predictor | Deviance | Residual df | Residual deviance | p-value |
| BMI change (for each 2 unit) | 7.23 | 10,756 | 1433.9 | **0.007** |
| BMI groups | 3.59 | 10,755 | 1430.3 | 0.058 |
| Sex (Female) | 11.06 | 10,754 | 1419.2 | **0.001** |
| Age | 8.35 | 10,753 | 1410.9 | **0.004** |
| BMI change vs BMI groups | 0.13 | 10,752 | 1410.8 | 0.723 |

*Model 1 included change in BMI, age at baseline as groups, sex, and baseline BMI (continuous), with an interaction between BMI change and age groups. **Model 2 included change in BMI, baseline BMI groups, sex, and age at baseline (continuous), with an interaction between BMI change and baseline BMI groups.


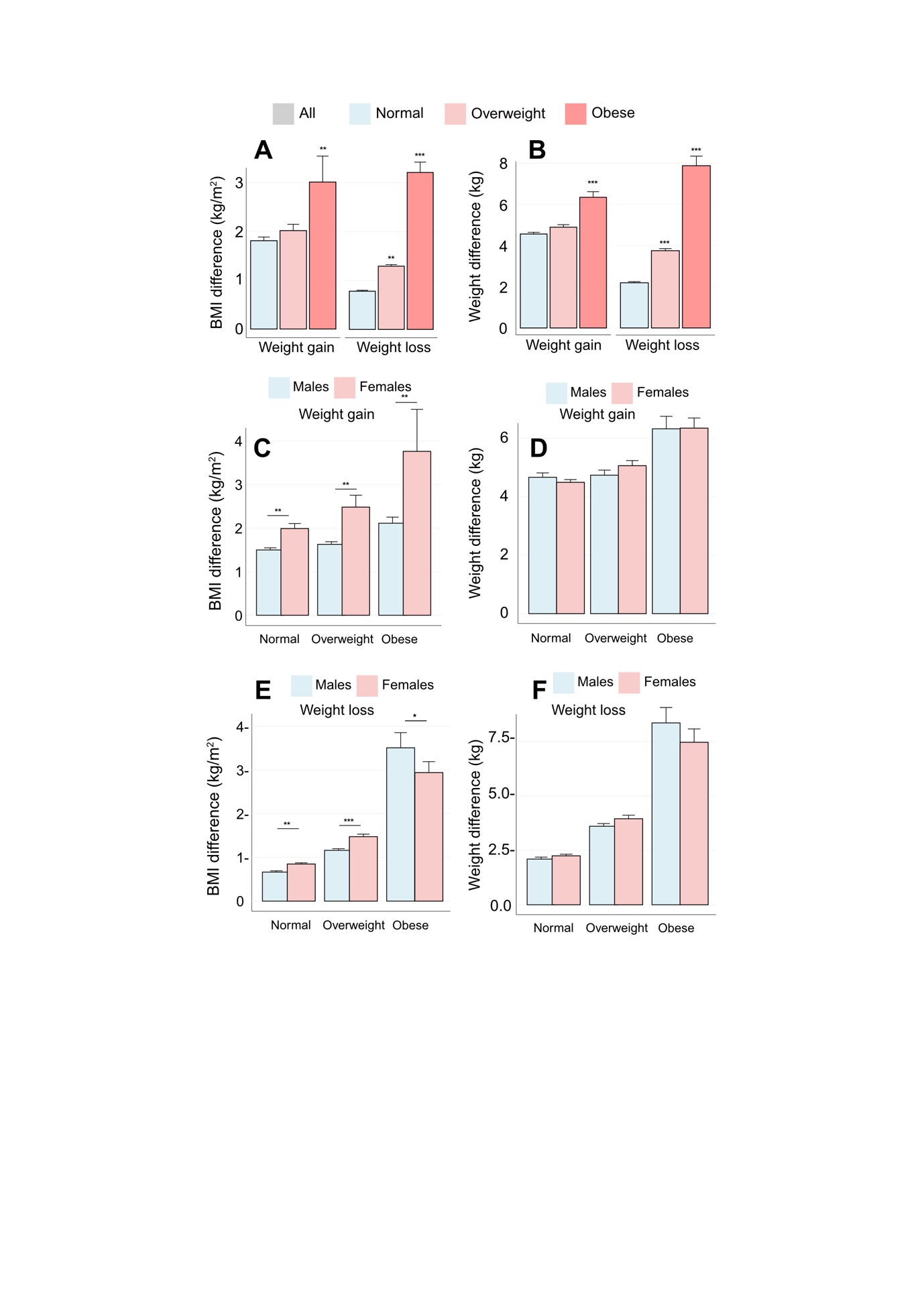

**Figure 1S**: Weight Change by BMI Category and Gender. (A) BMI change sorted by obesity categories at baseline and calculated among those who gained and lost weight separately. (B) BMI gain, among those who gained weight, sorted by gender and obesity at baseline. (C) Weight loss (kg), among those who lost weight, sorted by gender and obesity at baseline. (D) Weight change sorted by obesity categories at baseline and calculated among those who gained and lost weight separately (E) BMI gain grouped by gender and obesity categories at baseline. (F) Weight loss (kg) grouped by sex and obesity categories at baseline. Bars represent means, and error bars indicate confidence intervals (CI). Statistical differences were assessed using ANOVA or Student’s *t-*test for mean comparisons.


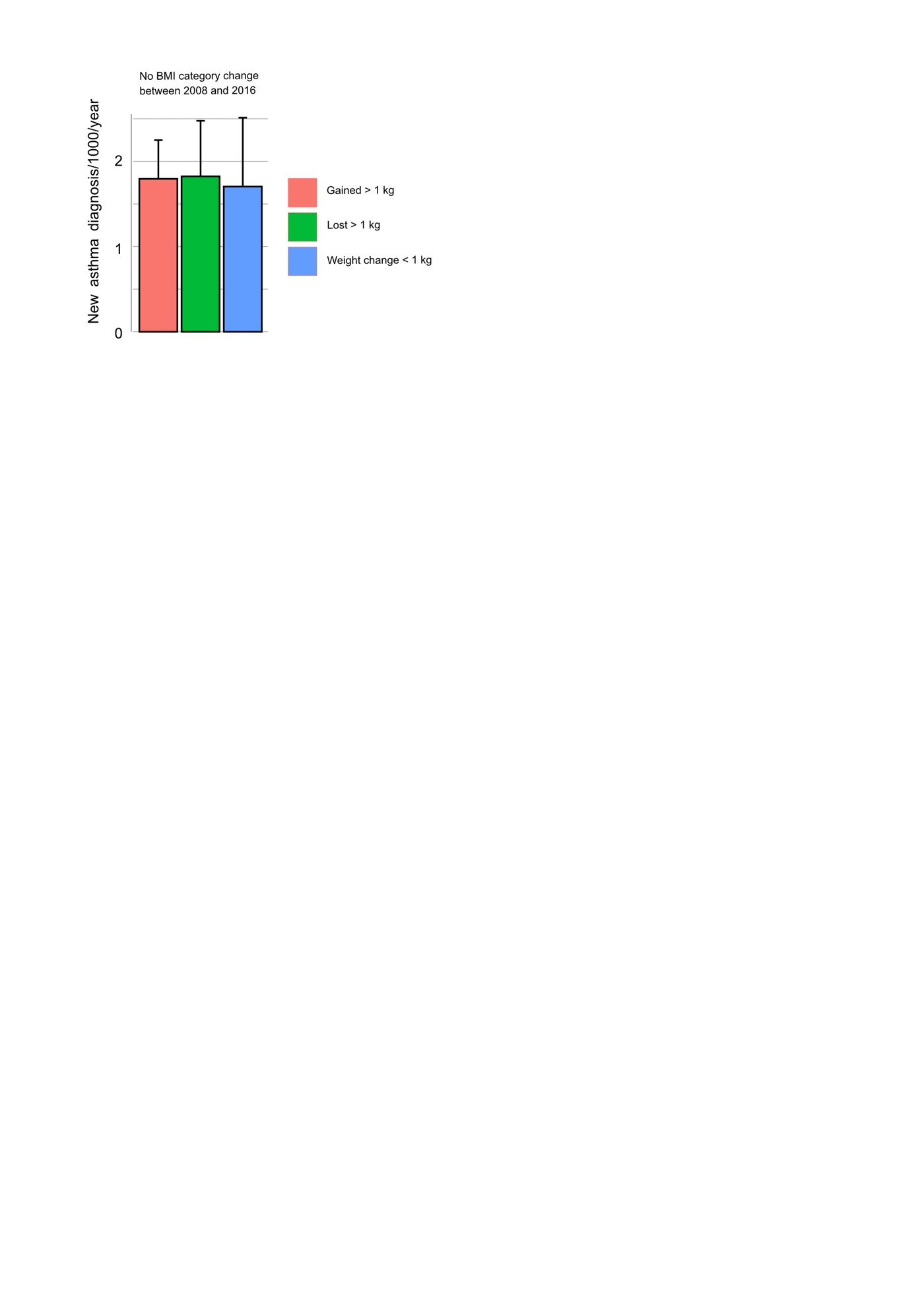


**Figure 2S:** Asthma incidence in those with stable BMI category.

**References:**

1. Nwaru BI, Ekerljung L, Rådinger M, et al. Cohort profile: the West Sweden Asthma Study (WSAS): a multidisciplinary population-based longitudinal study of asthma, allergy and respiratory conditions in adults. *BMJ Open*. Jun 19 2019;9(6):e027808. doi:10.1136/bmjopen-2018-027808

2. Rönmark E, Lundbäck B, Jönsson E, Jonsson AC, Lindström M, Sandström T. Incidence of asthma in adults--report from the Obstructive Lung Disease in Northern Sweden Study. *Allergy*. Nov 1997;52(11):1071-8. doi:10.1111/j.1398-9995.1997.tb00178.x

3. Bousquet J, Burney PG, Zuberbier T, et al. GA2LEN (Global Allergy and Asthma European Network) addresses the allergy and asthma 'epidemic'. *Allergy*. Jul 2009;64(7):969-77. doi:10.1111/j.1398-9995.2009.02059.x

4. Lindström M, Kotaniemi J, Jönsson E, Lundbäck B. Smoking, respiratory symptoms, and diseases : a comparative study between northern Sweden and northern Finland: report from the FinEsS study. *Chest*. Mar 2001;119(3):852-61. doi:10.1378/chest.119.3.852

5. Burney PG, Luczynska C, Chinn S, Jarvis D. The European Community Respiratory Health Survey. *Eur Respir J*. May 1994;7(5):954-60. doi:10.1183/09031936.94.07050954

6. Zhang J, Yu KF. What's the relative risk? A method of correcting the odds ratio in cohort studies of common outcomes. *Jama*. Nov 18 1998;280(19):1690-1. doi:10.1001/jama.280.19.1690
